# Oral bacteria and yeasts in relationship to oral ulcerations in hematopoietic stem cell transplant recipients

**DOI:** 10.1007/s00520-012-1463-2

**Published:** 2012-04-26

**Authors:** Alexa M. G. A. Laheij, Johannes J. de Soet, Peter A. von dem Borne, Ed J. Kuijper, Eefje A. Kraneveld, Cor van Loveren, Judith E. Raber-Durlacher

**Affiliations:** 1 Department of Preventive Dentistry, Academic Center for Dentistry Amsterdam (ACTA), University of Amsterdam and VU University, Gustav Mahlerlaan 3004, 1081 LA Amsterdam, The Netherlands; 2 Department of Hematology, Leiden University Medical Center, P.O. Box 9600, 2300 RC Leiden, The Netherlands; 3 Department of Medical Microbiology, Leiden University Medical Center, P.O. Box 9600, 2300 RC Leiden, The Netherlands

**Keywords:** Oral ulceration, HSCT, Periodontal pathogens, Yeasts, Oral mucositis, Stomatitis

## Abstract

**Background:**

Oral mucositis is a serious and debilitating side effect of conditioning regimens for hematopoietic stem cell transplant (HSCT). Through HSCT, the homeostasis in the oral cavity is disrupted. The contribution of the oral microflora to mucositis remains to be clarified. The aim of our study was to investigate the relationship between yeasts, bacteria associated with periodontitis, and oral ulcerations in HSCT recipients.

**Methods:**

This prospective observational study included 49 adult HSCT recipients. Twice weekly, oral ulcerations were scored, and oral rinsing samples were obtained. Samples were evaluated for the total bacterial load; the Gram-negative bacteria: *Aggregatibacter actinomycetemcomitans*, *Porphyromonas gingivalis*, *Prevotella intermedia*, *Parvimonas micra*, *Fusobacterium nucleatum*, *Tannerella forsythia*, and *Treponema denticola*; and the yeasts: *Candida albicans*, *Candida glabrata*, *Candida kefyr*, *Candida krusei*, *Candida parapsilosis*, and *Candida tropicalis* using real-time polymerase chain reaction with specific primers and probes. Explanatory variables for oral ulcerations were calculated using the multilevel generalized estimated equations (GEE) technique.

**Results:**

None of the samples was positive for *A. actinomycetemcomitans*, while *F. nucleatum* was found most often (66 % of samples). *C. albicans* was the most isolated yeast (88 % of samples), whereas *C. parapsilosis* was found in only 8 % of the samples. Multivariate GEE analyses identified *P. gingivalis*, *P. micra*, *T. denticola*, *F. nucleatum*, *C. glabrata*, and *C. kefyr* as significant explanatory variables of oral ulcerations.

**Conclusions:**

Our data indicate that *P. gingivalis* in particular, but also *P. micra*, *T. denticola*, *F. nucleatum*, *C. glabrata*, and *C. kefyr* may play a role in ulcerative oral mucositis in patients undergoing HSCT.

## Introduction

Mucositis remains one of the most common, serious, and painful side effects of cytotoxic cancer therapy [1]. It is reported that between 76 % and 89 % of all patients receiving a myeloablative hematopoietic stem cell transplant (HSCT) experience oral mucositis [2–4]. Mucositis can be so painful that patients are not able to eat or drink, and it can result in a poorer treatment outcome [5].

The pathobiology of mucositis consists of five interdependent stages: initiation, the primary damage response (messaging and signaling), amplification of damage responses, ulceration, and finally, healing of the ulcers. Oral microorganisms are thought to be involved in the ulceration phase, where they probably are able to intensify the inflammatory process and aggravate or promote the formation of ulcers [6].

Most of the bacterial species present in the oral cavity are harmless commensal bacteria, and under normal healthy conditions, there is homeostasis in the oral cavity. In patients with cancer, this delicate balance can be disturbed by the cancer itself, the anti-cancer treatment, or by the supportive therapies that all may contribute to a shift in the oral microflora of the oral cavity from mainly Gram-positive to Gram-negative bacteria [7]. This disruption of the balance may be related to direct cytotoxic effect on the oral flora, granulocytopenia, altered salivary output, alteration in cytokine release, use of antibiotics, compromised oral hygiene, and the acquisition of hospital-associated pathogens [8]. In the cell wall of Gram-negative obligatory or facultative anaerobic bacteria including *Actinomyces actinomycetemcomitans*, *Fusobacterium nucleatum*, *Porphyromonas gingivalis*, *Parvimonas micra*, *Prevotella intermedia*, *Treponema denticola*, and *Tannerella forsythia*, components such as the endotoxin lipopolysaccharide (LPS) are present. LPS activates macrophages to produce inflammatory mediators like interleukin 1 (IL-1), interleukin 6 (IL-6), tumor necrosis factor-alpha (TNF-α), prostaglandin E2 (PGE_2_), and matrix metalloproteinases (MMPs) [9]. These bacteria are associated with periodontitis and gingivitis, and they may have the ability to aggravate the inflammatory process in mucositis.

In addition, oral microbes and cytokines may enter the bloodstream when the integrity of the oral mucosal barrier is disrupted and may induce fever and infectious complications including sepsis [10–12]. Periodontal infections are also associated with fever and sepsis in patients treated with high-dose chemotherapy [13]. However, until now, it is unclear whether there is an association between periodontal pathogens and mucositis [8].

*Candida* species and particularly *Candida albicans* are also part of the commensal oral flora in a large part of the population. Between 20 % and 75 % of individuals in the general population is colonized with *Candida* species [14]. When the oral homeostasis is disrupted, *Candida* species may overgrow and cause local oral infection (candidiasis). Candidiasis can cause systemic infections and contributes highly to morbidity in infected patients [15]. Risk factors for oral candidiasis are immunosuppression, hyposalivation, local tissue damage, higher age, and wearing dentures [16–18]. HSCT recipients have several of these risk factors and are therefore at high risk for developing candidiasis and invasive *Candida* infection (candidemias). In a systematic review, oral yeast colonization is reported to occur in 73 % of patients treated with high-dose chemotherapy, and candidiasis develops in 38 % of these patients [16].

In addition to infections caused by *C. albicans*, infections may be caused by non-albicans *Candida* species such as *Candida glabrata*, *Candida parapsilosis*, *Candida tropicalis*, *Candida krusei*, and *Candida kefyr* as well [19]. Non-albicans *Candida* species are increasing and are estimated to account for 40–70 % of today's systemic *Candida* infections in HSCT recipients with hematological malignancies. Risk factors for candidemias due to *C. tropicalis* and *C. krusei* include neutropenia and HSCT [20]. Although seldom found, *C. kefyr* has been linked to bloodstream infections in patients with hematological malignancies [21, 22]. A recently published study found an association between *C. albicans* and oral mucositis in patients receiving high-dose chemotherapy for hematological malignancies [23].

To the best of our knowledge, no studies have been performed in which HSCT patients with and without ulcerations were prospectively followed over time, and bacterial and fungal analysis of standardized samples was carried out using sensitive real-time polymerase chain reaction (PCR). The aim of this prospective, observational study was to investigate the relationship between bacteria associated with periodontitis, yeasts, and oral ulcerations in HSCT recipients using real-time PCR. Data on the relationship between several patient characteristics, herpes viruses, and oral ulcerations in the same patient group have been published elsewhere [24].

## Patients and methods

### Patients

Forty-nine adult patients that underwent HSCT were enrolled in this study. All patients were treated for hematological malignancies at the Leiden University Medical Center between November 2006 and June 2009. The study was approved by the Medical Ethical Committee of the Leiden University Medical Center, and patients gave their written informed consent.

T-cell-depleted stem cell transplantation was performed either with a myeloablative conditioning (MAC) or a reduced intensity-conditioning regimen (RIC) [25–27]. During granulocytopenia, all patients received oral digestive tract decontamination with oral neomycin, polymyxin, ciprofloxacin, and a neomycin–polymyxin paste or rinse. Antifungal prophylaxis consisted of oral amphotericin B tablets in combination with lozenges to be sucked on slowly. Antistreptococcal prophylaxis with intravenous benzylpenicillin was given 7 days following MAC and continued for 14 days. Pre-emptive antibiotics, consisting of vancomycin and ceftazidime, were administered when patients developed a fever. During hospitalization, all patients received standardized oral care aimed at preventing the accumulation of plaque and keeping the oral tissues moist.

### Oral assessment and sampling

An oral assessment was performed at least twice weekly starting before or as soon as possible after the administration of the conditioning regimen until hospital discharge. Oral mucositis was scored according to the criteria of the World Health Organization [28] at eight non-keratinized anatomical sites (labial and buccal mucosa, floor of mouth, lateral and ventral tongue, and soft palate) by one trained dentist (J.E.R-D). Ulcerations present at the keratinized/specialized mucosa (vermillion borders of the lip, gingiva, dorsum of the tongue, and hard palate) were noted separately.

Oral rinsing samples were taken at each oral assessment using 10 ml of 0.9 % sterile saline solution. Patients were asked to rinse for 30 s. Samples were frozen at −20°C within 3 h and thereafter stored at −80°C until analysis.

### Bacterial and yeast load determination

A total number of 233 rinsing samples were collected, for 90 % of patients varying between two to eight samples. All samples were thawed and concentrated ten times by freeze-drying 1 ml of sample and dissolving the residuum in 100 μl of PCR water (Roche Diagnostics, Almere, The Netherlands). DNA was isolated from all samples with the MagNa Pure LC DNA Isolation Kit III for bacteria and fungi (Roche Diagnostics) using the MagNa Pure LC 2.0 instrument (Roche Diagnostics). Bacterial and yeast loads were determined in duplo by real-time PCR using the LightCycler® 480-II Instrument (Roche Diagnostics).

Loads of *A. actinomycetemcomitans* and *F. nucleatum* (reaction A); *P. gingivalis* and *T. forsythia* (reaction B); *P. micra*, *P. intermedia*, and *T. denticola* (reaction C); and *C. albicans* and PhHV [36] (reaction D) were determined by multiplex real-time PCR. PhHV served as an internal control for DNA extraction and PCR inhibition. Loads of *C. glabrata*, *C. kefyr*, *C. krusei*, *C. parapsilosis*, *C. tropicalis*, and the total bacterial load were determined by monoplex real-time PCR (Table 1). All PCRs were carried out in 20-μl reaction volume containing 10 μl of LightCycler® 480 Probes Master (Roche Diagnostics).

**Table 1:** Details of real-time PCR reactions for the detection of bacteria and yeasts

	Sequence (5′ → 3′)		Amount added to PCR reaction (pmol)	Amount of sample DNA added to PCR reaction	Reference
Bacteria					
*A. actinomycetemcomitans*	Forward	GAA CCT TAC CTA CTC TTG ACA TCC GAA	1.8	4^a^	[30]
	Reverse	TGC AGC ACC TGT CTC AAA GC	1.8		
	Probe	FAM-AGA ACT CAG AGA TGG GTT TGT GCC TTA GGG-BBQ	0.4		
*P. gingivalis*	Forward	GCG CTC AAC GTT CAG CC	1.8	4^a^	[29]
	Reverse	CAC GAA TTC CGC CTGC	1.8		
	Probe	Cyan500-CAC TGA ACT CAA GCC CGG CAG TTT CAA-BBQ	0.4		
*P. intermedia*	Forward	CGG TCT GTT AAG CGT GTT GTG	1.8	4^a^	[30]
	Reverse	CAC CAT GAA TTC CGC ATA CG	1.8		
	Probe	Cyan500-TGG CGG ACT TGA GTG CAC GC-BBQ	0.4		
*T. forsythia*	Forward	GGG TGA GTA ACG CGT ATG TAA CCT	1.8	4^a^	[32]
	Reverse	ACC CAT CCG CAA CCA ATA AA	1.8		
	Probe	YAK-CCC GCA ACA GAG GGA TAA CCC GC-BBQ	0.4		
*P. micra*	Forward	GCC GTA AAC GAT GAG TGC TAG G	1.8	4^a^	[30]
	Reverse	CCA GGC GGA ATG CTT AGT GT	1.8		
	Probe	YAK-TGG GAG TCA AAT CTC GGT GGC G-BBQ	0.4		
*F. nucleatum*	Forward	GGA TTT ATT GGG CGT AAA GC	1.8	4^a^	Modified to [30]
	Reverse	GGC ATT CCT ACA AAT ATC TAC GAA	1.8		
	Probe	LC610-TTC ACC TCT ACA CTT GTA GTT CCG CTT-BBQ	0.4		
*T. denticola*	Forward	CCG AAT GTG CTC ATT TAC ATA AAG GT	1.8	4^a^	Modified to [31]
	Reverse	GAT ACC CAT CGT TGC CTT GGT	1.8		
	Probe	LC670-CTC ACC AAC TAG CTA ATG GGA CGC GG-BBQ	0.4		
Universal bacterial load	Forward	TCC TAC GGG AGG CAG CAG T	7.5	2	[33]
	Reverse	GGA CTA CCA GGG TAT CTA ATC CTG TT	7.5		
	Probe	FAM-CGT ATT ACC GCG GCT GCT GGC AC-BBQ	0.38		
Yeasts					
*C. albicans*	Forward	GGG TTT GCT TGA AAG ACG GTA	24	3^a^	[34]
	Reverse	TTG AAG ATA TAC GTG GTG GAC GTT A	24		
	Probe	FAM-ACC TAA GCC ATT GTC AAA GCG ATC CCG-TAMRA	2.5		
*C. glabrata*	Forward	TTT CTC CTG CCT GCG CTT AA	10	3	[34]
	Reverse	ACG CAC ACT CCC AGG TCT TT	10		
	Probe	FAM-AGA ACA CCC ACC AAC CGC GCA-TAMRA	0.4		
*C. tropicalis*	Forward	GCG TCA TTT CTC CCT CAA ACC	10	3	[35]
	Reverse	TGG CCA CTA GCA AAA TAA GCG T	10		
	Probe	FAM-CGG GTT TGG TGT TGA GCA ATA CGC TAG G-TAMRA	0.4		
*C. kefyr*	Forward	GGC TGC GTG TCG AGT CTA TG	10	3	[34]
	Reverse	TGA CCC AAG CTT ACC ACG AAT	10		
	Probe	FAM-ACT CTT GCA CAT CTA CGT CTT AGG TTT GCG C-BBQ	0.4		
*C. krusei*	Forward	GCT GCG ACT CGC CTG AA	10	3	[34]
	Reverse	TTG TCT CGC AAC ACT CGC TCT	10		
	Probe	FAM-CTA GTT CGC TCG GCC AGC TTC GCT-BBQ	0.4		
*C. parapsilosis*	Forward	GGG TTT GGT GTT GAG CGA TAC	10	3	[34]
	Reverse	GGA GTT TGT ACC AAT GAG TGG AAA	10		
	Probe	FAM-CTC CGC CTT TCT TTC AAG CAA ACC CAG-BBQ	0.4		

^a^Total amount of sample DNA for the multiplex PCR reaction

The samples for bacterial analysis were subjected to pre-incubation cycle of 95°C for 5 min, followed by 45 cycles of quantification at 95°C for 10 s and at 60°C for 20 s. The samples for *Candida* analysis were subjected to a pre-incubation cycle of 95°C for 10 min, followed by 50 cycles of quantification at 95°C for 10 s, at 60°C for 30 s, and at 72°C for 30 s.

Serial tenfold dilutions of homologous DNA were used as standard curves. For quantification, the results of the samples were calculated using the standard curve of the corresponding bacterium or yeast. For quantification of the total bacterial load, a standard curve of *A. actinomycetemcomitans* was used. PCR water was used as a negative control in every run.

### Statistics

Loads are shown as CFU per milliliter of rinsing sample. To adjust for the unreliability of low positive signals in real-time PCR, the loads of these samples were set at 80 % of the detection limit of the corresponding bacterium or yeast. To adjust for skewness of the data, ^10^log transformed bacterial and yeast loads were used in the analysis. Mucositis scores were recalculated into binary scores: mucositis grade 0–1 was scored as no ulceration present, and mucositis grades 2–4 were scored as ulceration present. It was not possible to look at different grades of mucositis as a dependent variable since some mucositis scores did not occur frequently enough. To be able to look at shifts in the oral cavity, the absolute loads of the bacterial and *Candida* species were recalculated into percentages/ratio in relation to the total bacterial load.

Explanatory variables of oral ulcerations were calculated using the multilevel binary logistic regression procedure called the generalized estimated equations (GEE) technique, with first order autoregressive correlation structure and a robust estimation procedure. Independent variables were screened as possible explanatory variables for oral ulcerations using univariate GEE analyses. An independent variable with a *p* value <0.20 was entered in the multivariate GEE analysis. A *p* value <0.05 was considered statistically significant. All statistical analyses were calculated using SPSS version 18.0.

## Results

### Patient characteristics and oral assessment outcomes

The characteristics of the 49 patients that participated in this study are summarized in Table 2. At 232 (out of 233) time points, WHO scores involving non-keratinized oral sites were recorded. Most of the patients (70 %) developed mucositis during their stay in the hospital. One patient developed mucositis grade 1 at most, 23 patients experienced peak mucositis of grade 2, seven patients developed ultimately grade 3 mucositis, and three patients suffered from a maximum of grade 4 mucositis. Furthermore, 25 patients (51 %) developed an ulceration on the keratinized/specialized mucosa. At 226 (out of 233) time points, ulcerations on the keratinized mucosa were scored.

**Table 2:** Patient characteristics

Number of patients	49
Male	27 (55 %)
Female	22 (44 %)
Age (mean ± SD)	48.8 (±13.6) years
Diagnosis	
Acute myeloid leukemia	19 (39 %)
Multiple myeloma	10 (20 %)
Acute lymphoblastic leukemia	5 (10 %)
Non-Hodgkin lymphoma	5 (10 %)
Myelodysplastic syndrome	3 (6 %)
Chronic myeloid leukemia	2 (4 %)
Hodgkin lymphoma	2 (4 %)
Chronic lymphocytic leukemia	2 (4 %)
Others	1 (2 %)
Donor type	
Matched sibling	17 (35 %)
Matched unrelated	25 (57 %)
Others	4 (8 %)
Conditioning regimen	
Myeloablative	25 (51 %)
Reduced intensity	24 (49 %)
Length of stay in the hospital (mean ± SD)	27 (±8.9) days

### Oral bacterial load

The percentages of positive samples and positive patients and the measured ranges for the respective bacteria are shown in Table 3. *A. actinomycetemcomitans* could not be found in any of the samples. On the other hand, *F. nucleatum* was the bacterium that was found most often. All samples were positive for bacteria. The total load of periodontal bacteria as a proportion of the total bacterial load was 1.2 % on average. In about 84 % of the samples, the proportion of periodontal bacteria was lower than 1 %. In six samples, the proportion of periodontal bacteria was higher than 10 %, with a maximum of 63 %.

**Table 3:** Descriptives of the determined bacterial species

Bacterial species	Percentage positive samples	Percentage positive patients	Range (^10^log CFU/ml)
*A. actinomycetemcomitans*	0	0	0
*P. gingivalis*	11 %	20 %	4.15–5.85
*P. intermedia*	8 %	18 %	2.20–3.53
*T. forsythia*	31 %	48 %	1.82–5.02
*P. micra*	43 %	63 %	2.23–5.39
*F. nucleatum*	66 %	86 %	3.23–5.63
*T. denticola*	15 %	25 %	3.65–5.94
Total bacterial load	100 %	100 %	4.0–8.38

### *Candida* loads

All patients had samples that were positive for a least one *Candida* species during treatment. Only 4 % of the samples were negative for *Candida* species. Most patients were positive for a maximum of four different *Candida* species. The percentages of positive samples, patients, and the measured ranges of *Candida* loads are shown in Table 4. *C. albicans* was found most often, whereas *C. parapsilosis* was found the least.

**Table 4:** Descriptive properties of the determined *Candida* species

Yeasts	Percentage positive samples	Percentage positive patients	Range (^10^log CFU/ml)
*C. albicans*	88 %	94 %	1.08–5.57
*C. glabrata*	54 %	70 %	2.57–4.12
*C. tropicalis*	58 %	80 %	1.86–4.44
*C. kefyr*	56 %	94 %	1.18–1.57
*C. krusei*	26 %	67 %	2.40–5.48
*C. parapsilosis*	8 %	25 %	3.18–3.15

In relationship to the total bacterial load, the ratio of the total *Candida* load was on average 2 %. There was no significant correlation between the total bacterial load and the total *Candida* load (Pearson's rho = 0.026, *p* = 0.718).

### Explanatory variables of oral ulcerations on non-keratinized mucosa

The bacterial and *Candida* species were represented as explanatory variables in three different ways. First, the presence or absence for each species was determined and entered in the GEE analyses. The univariate analyses are depicted in the second and third columns of Table 5. Independent variables with a *p* value <0.20 in the univariate analyses were entered together in the multivariate analysis. When *P. gingivalis* or *C. kefyr* was present, the chance of having an ulceration on the non-keratinized mucosa was significantly higher.

**Table 5:** Uni- and multivariate GEE analyses of predictors for ulcerations on the oral non-keratinized mucosa

Independent variable	Univariate analysis		Multivariate analysis	
	*p* value	exp(B)	*p* value	exp(B)
Presence *P. gingivalis*	0.078	1.98	0.007*	3.36
Presence *P. intermedia*	0.378	1.35		
Presence *T. forsythia*	0.022	0.45	0.115	0.48
Presence *P. micra*	0.124	0.65	0.333	0.75
Presence *F. nucleatum*	0.118	0.52	0.377	0.612
Presence *T. denticola*	0.378	0.72		
Presence *C. albicans*	0.331	1.38		
Presence *C. glabrata*	0.969	0.99		
Presence *C. kefyr*	0.048	1.91	0.029*	2.01
Presence *C. tropicalis*	0.492	1.18		
Presence *C. krusei*	0.422	1.38		
Presence *C. parapsilosis*	^a^			
Load *P. gingivalis*	0.037	1.17	0.004*	1.37
Load *P. intermedia*	0.086	1.23	0.355	1.22
Load *T. forsythia*	0.103	0.85	0.073	0.77
Load *P. micra*	0.068	0.82	0.056	0.78
Load *F. nucleatum*	0.402	0.90		
Load *T. denticola*	0.559	0.94		
Load *C. albicans*	0.473	0.93		
Load *C. glabrata*	0.883	0.98		
Load *C. kefyr*	0.055	1.78	0.013*	2.056
Load *C. tropicalis*	0.396	1.13		
Load *C. krusei*	0.336	1.16		
Load *C. parapsilosis*	^a^			
Total bacterial load	0.254	0.86		
Total *Candida* load	0.975	1.00		
Percentage *P. gingivalis*	0.002	1.16	0.001*	1.372
Percentage *P. intermedia*	0.852	0.46		
Percentage *T. forsythia*	0.563	0.94		
Percentage *P. micra*	0.059	0.11	0.001*	0.00
Percentage *F. nucleatum*	0.099	1.44	0.015*	1.58
Percentage *T. denticola*	0.000	0.98	0.000*	0.87
Percentage *C. albicans*	0.143	1.07	0.080	1.07
Percentage *C. glabrata*	0.081	1.65	0.000*	3.49
Percentage *C. kefyr*	0.366			
Percentage *C. tropicalis*	0.297	1.24		
Percentage *C. krusei*	^a^			
Percentage *C. parapsilosis*	^a^			

*Significant (*p* < 0.05)

^a^Could not be determined because of insufficient power for the analysis of small strata

Secondly, the absolute loads of all species were entered in the GEE analyses (see Table 5). The results of the multivariate analysis showed that also the load of *P. gingivalis* and *C. kefyr* were significant explanatory variables. The higher the load of *P. gingivalis* or *C. kefyr*, the higher the chance of an ulceration.

Thirdly, the percentages of bacterial and proportion of *Candida* species in relation to the total bacterial load were entered in the GEE analyses (see Table 5). In the multivariate analysis, the percentages of *P. gingivalis*, *P. micra*, *T. denticola*, *F. nucleatum*, and proportion of *C. glabrata* in relation to the total bacterial load turned out to be significant explanatory variables of oral ulcerations on the non-keratinized mucosa. The total bacterial and total *Candida* load were no significant explanatory variables of oral ulcerations on the non-keratinized mucosa.

### Explanatory variables of oral ulcerations on keratinized mucosa

The bacterial and *Candida* species were represented as explanatory variables in the same way as for the analyses regarding ulcerations of the non-keratinized mucosa. The results of the GEE analyses are shown in Table 6. Looking at the presence of bacterial and *Candida* species in the multivariate analysis, *P. gingivalis*, *P. micra*, and *C. kefyr* were identified as significant explanatory variables of oral ulcerations. Also, the load of *P. gingivalis* and *C. kefyr* were significant explanatory variables. However, when the percentages of bacteria and proportions of *Candida* species in relation to the total bacterial load were considered, none of the possible explanatory species were significant in the multivariate GEE analysis. The total bacterial and total *Candida* load were no significant explanatory variables of oral ulcerations on the keratinized mucosa as well.

**Table 6:** Uni- and multivariate GEE analyses of predictors of ulcerations on the oral keratinized mucosa

Independent variable	Univariate analysis		Multivariate analysis	
	*p* value	exp(B)	*p* value	exp(B)
Presence *P. gingivalis*	0.007	3.293	0.005*	4.38
Presence *P. intermedia*	0.238	2.22		
Presence *T. forsythia*	0.741	0.923		
Presence *P. micra*	0.077	0.54	0.043*	0.46
Presence *F. nucleatum*	0.657	0.854		
Presence *T. denticola*	0.924	0.959		
Presence *C. albicans*	0.539	0.746		
Presence *C. glabrata*	0.722	0.863		
Presence *C. kefyr*	0.082	0.587	0.005*	0.53
Presence *C. tropicalis*	0.115	0.619	0.235	0.68
Presence *C. krusei*	0.788	1.08		
Presence *C. parapsilosis*	^a^			
Load *P. gingivalis*	0.011	1.29	0.034*	0.75
Load *P. intermedia*	0.134	1.48	0.532	0.79
Load *T. forsythia*	0.915	1.01		
Load *P. micra*	0.195	0.84	0.094	1.29
Load *F. nucleatum*	0.611	0.95		
Load *T. denticola*	0.924	1.01		
Load *C. albicans*	0.368	0.85		
Load *C. glabrata*	0.883	0.97		
Load *C. kefyr*	0.047	0.57	0.028*	1.83
Load *C. krusei*	0.728	1.04		
Load *C. tropicalis*	0.240	0.84		
Load *C. parapsilosis*	^a^			
Total bacterial load	0.829	1.02		
Total *Candida* load	0.526	0.89		
Percentage *P. gingivalis*	0.514	0.94		
Percentage *P. intermedia*	0.846	2.57		
Percentage *T. forsythia*	0.979	1.00		
Percentage *P. micra*	0.715	0.46		
Percentage *F. nucleatum*	0.092	1.64	0.162	1.76
Percentage *T. denticola*	0.117	1.06		
Percentage *C. albicans*	0.087	1.08	0.416	1.07
Percentage *C. glabrata*	0.096	1.66	0.119	1.70
Percentage *C. kefyr*	0.606	266.11		
Percentage *C. tropicalis*	0.307	2.14		
Percentage *C. krusei*	0.602	0.19		
Percentage *C. parapsilosis*	0.080	1.22	0.646	0.92

*Significant (*p* < 0.05)

^a^Could not be determined because of insufficient power for the analysis of small strata

## Discussion

The results of our study suggest that there is a relationship between several bacteria associated with periodontitis, yeasts, and oral ulcerations in HSCT patients. The most striking result was that the Gram-negative anaerobic bacterium *P. gingivalis* was a constant explanatory variable and had a positive predictive value for oral ulcerations. In addition, the anaerobic bacteria *P. micra*, *F. nucleatum*, and *T. denticola* and the yeasts *C. glabrata* and *C. kefyr* seemed to be related to oral ulcerations as well.

The explanatory variables for ulcerations were calculated in three different ways, since microorganisms can be involved in infection or disease in different ways. Some microorganisms cause disease in small numbers, whereas other microorganisms need to be present in high numbers to provoke an infection. In addition, a shift in bacterial composition or the overgrowth of yeasts may be responsible for disease.

The GEE analysis is a multilevel regression method to determine the effect that independent variables have on dependent variables. The explanatory (independent) variables cannot be seen as causative factors. They explain, but do not cause the dependent variable, in this case oral ulcerations. Causative variables need to be present in time before the dependent variables. During the time of our study, a pre-transplant oral evaluation and sampling, including periodontal probing, was not part of the standard of care. Therefore, it was not possible to make such conclusions from this study.

No clear pattern or association between mucositis and the oral microflora emerges from literature [8]. However, it is difficult to compare studies since they differ on the populations that were studied, the chemotherapeutic regimens administered, the sampling and sample analysis methods, the microorganisms studied, the collection times, and the scoring methods for mucositis. Thus, it is difficult to compare our results to those of other studies. Moreover, the bacteria that we studied were not described previously in relation to mucositis. *A. actinomycetemcomitans*, *P. gingivalis*, *Prevotella* spp., and *F. nucleatum* were studied in children receiving chemotherapy, but none of these species was isolated [37]. The absence of *A. actinomycetemcomitans* in the present population is remarkable and may be due to the antibiotics used. However, in periodontitis patients, *A. actinomycetemcomitans* is usually not eradicated by these antibiotics. Approximately 15–30 % of the normal population is positive for *A. actinomycetemcomitans*; thus, patients positive for *A. actinomycetemcomitans* were expected in our patient group as well. Although it cannot be excluded that *A. actinomycetemcomitans* was still present in subgingival biofilms, we do not have a good explanation for the absence of *A. actinomycetemcomitans* in the rinsing samples obtained from our patient group.

*P. gingivalis* was consistently associated with oral ulcerations in the present study and had a positive predictive value. *P. gingivalis* is present in 10–25 % of healthy subjects and 53–90 % in periodontitis patients [38, 39]. It possesses several virulence factors like fimbriae that enables the bacterium to attach to and invade into epithelial cells, the expression of proteases [40], and LPS and a polysaccharide capsule that are both highly antigenic and can induce the production of pro-inflammatory cytokines [41, 42]. These virulence factors might be able to prolong or intensify oral ulcerations or impair wound healing and that could explain the role of *P. gingivalis* in mucositis.

In addition, *P. gingivalis* may play a role in the initiation phase of mucositis. Chronic exposure to *P. gingivalis* may upregulate the expression of Toll-like receptors (TLR) on epithelial and endothelial cells in the oral mucosa. Increased expression of TLR may accelerate the initiation of mucositis when “CRAMPs” (endogenous damage-associated pattern molecules released by cells damaged by chemoradiation) bind to TLR resulting in activation of nuclear factor kappa-B (NF-κB) [43].

The role of herpes viruses in this patient group was described elsewhere [24]. There was no association between HSV-1 and *P. gingivalis* in these patients. So, the mechanism in which both microorganisms are involved in oral mucositis is thought to be different. Reactivation of herpes simplex virus type-I (HSV-1) in the oral cavity most probably causes oral ulcerations in HSCT patients, while *P. gingivalis* is supposed to be involved in the onset of mucositis and in maintaining or intensifying oral ulcerations.

We found high levels of colonization for all *Candida* species except for *C. parapsilosis*. The levels that we found were higher than those reported previously for *C. albicans*, and much higher for *C. glabrata*, *C. tropicalis*, *C krusei*, *C. kefyr*, and *C. parapsilosis* in patients with hematological malignancies [17, 19]. Most patients were colonized with as much as four different *Candida* species. Others found at most two different yeast species in oral samples [18]. Most previous studies used traditional culturing methods, while we used sensitive real-time PCR. The detection level for *Candida* species in our samples was very low, also because we concentrated the samples ten times. The method we used probably explains the high level of colonization that we found. Furthermore, it is feasible that patients in this hospital have a high degree of colonization.

Only few studies link *Candida* species with oral mucositis or ulcerations in HSCT recipients [23, 44]. *C. glabrata* and *C. kefyr* have not been described as being associated to oral ulcerations before. A recently published study found an association between *C. albicans* and oral mucositis in patients receiving chemotherapy for hematological malignancies. In contrast to our study, in this study, patient samples in the absence of mucositis were not studied [23]. In children with acute lymphoblastic leukemia, an association between oral mucositis and *Candida* species has been found as well [44].

Although *C. kefyr* is considered to be a rare species, we found a very high colonization rate in our patient group (94 % of patients). Interestingly, the load in almost all samples was around the detection limit. Data on *C. kefyr* colonization are scarce; one study describes the high colonization rate of *C. kefyr* in patients with hematological malignancies [22]. Furthermore, there are case reports describing bloodstream infections with *C. kefyr* [21, 22], though invasive infections with this species are very rare at the LUMC. Most previous studies used traditional culturing methods, which are less sensitive than real-time PCR we have been using, and low loads could have been missed. It is also feasible that the primers and probe for *C. kefyr* reacted with another yeast as well. However, cross-reactivity tests in our lab did not reveal cross-reactivity of our primer-probe set with the other *Candida* species (*C. albicans*, *C. glabrata*, *C. krusei*, *C. parapsilosis*, *C. tropicalis*, and *Candida dubliniensis*). At the time of analysis in the laboratory, a search with the Basic Local Alignment Search Tool (BLAST) did not reveal cross reactivity with other yeasts as well. Thus, it seems plausible that this patient group had a high level of colonization with low loads of *C. kefyr*.

Despite anti-fungal and antibacterial prophylaxis, all patients were positive for *Candida* species, and bacterial loads were quite high. Resistance of *Candida* to amphotericin B, which was used prophylactically by our patient population, was probably not the reason why we found high levels of colonization, since most *Candida* species are not resistant to amphotericin B. However, some strains of *C. krusei* and *C. kefyr* have been reported to display resistance to amphotericin B [20]. A likely explanation may be a low compliance to the prophylactic scheme, since patients reported having difficulty sucking on the amphotericin B lozenges, particularly when having a dry mouth.

We were not surprised to find high numbers of bacteria despite antibiotic prophylaxis. Bacteria in an oral biofilm are difficult to eradicate with systemically administered antibiotics because the biofilm highly protects them against an attack. Furthermore, the bacteria in our study were relatively insensitive to the antibiotic prophylaxis used, and therefore, the bacterial load of the less sensitive organisms may become higher. Patients that developed a fever during neutropenia received an additional treatment with antibiotics that may have influenced the composition of the oral flora that we studied. In spite of all these antibiotic treatments, patients developed (ulcerative) mucositis, and the association with a part of the oral microflora was found.

In literature, there are conflicting results from studies that used antimicrobials to prevent oral mucositis [7]. These conflicting results may be due to the selection of inappropriate antimicrobials for the flora that is important to be eliminated (e.g., anaerobic Gram-negative bacteria), and the way of administering the antimicrobials (e.g., systemically versus locally) may affect the results as well.

In conclusion, *P. gingivalis*, in particular, but also *P. micra*, *F. nucleatum*, *T. denticola*, and the yeasts *C. glabrata* and *C. kefyr* are explanatory variables and have a positive predictive value for mucosal ulcerations in HSCT patients. Future studies on the role of these microorganisms in mucositis are warranted.
